# LncRNA HULC as a potential predictor of prognosis and clinicopathological features in patients with digestive system tumors: a meta-analysis

**DOI:** 10.18632/aging.203903

**Published:** 2022-02-18

**Authors:** Duo Li, Rui Wang, Na Wu, Yongqiang Yu

**Affiliations:** 1 Department of Gastroenterology, The First Affiliated Hospital of Hebei North University, Zhangjiakou, Hebei, China

**Keywords:** lncRNA, HULC, digestive system cancer, prognosis

## Abstract

Objective: This meta-analysis aimed to evaluate the correlation between lncRNA HULC, prognosis and clinicopathological characteristics in patients with digestive system tumors.

Methods: The relevant literatures were collected through PubMed, Web of Science and Embase up to February 2021. Hazard ratios (HRs) and 95% confidence intervals (CIs) were calculated to assess the prognostic value of HULC in patients with digestive system tumors. The clinicopathological characteristics of HULC in patients were estimated by odds ratios (ORs).

Results: A total of 14 studies involving 1312 patients were included. The up-regulated expression level of HULC was associated with poorer overall survival (OS) in patients with digestive system tumors (HR = 1.83, 95% CI: 1.05-3.19, *P* = 0.033). Subgroup analysis showed that cancer type (pancreatic cancer or gastric cancer), residence region (China, Japan or Korea), and specimen (serum) significantly associated between HULC and OS. In addition, high HULC expression significantly increased the risk of high TNM stage (OR = 2.51, 95%CI: 1.36-4.62, *P* < 0.05), poor differentiation (OR = 1.38, 95%CI: 1.02-1.87, *P* < 0.05) and lymphatic node metastasis (LNM, OR = 4.93, 95% CI: 3.47-6.99, *P* < 0.05).

Conclusions: High expression level of HULC is related to OS, TNM stage, differentiation and LNM. Therefore, HULC can be used as a new potential predictor for prognosis and clinicopathological features of patients with digestive system tumors.

## INTRODUCTION

Digestive system tumor is a heterogeneous group of complex diseases affecting different organs, and the vast majority of it is malignancies [1, 2]. Digestive system tumor is the common cause of cancer deaths [3, 4]. According to World Health Organization (WHO) classification of tumors, digestive system tumors include esophageal cancer, gastric cancer, small intestine cancer, hepatocellular carcinoma, gallbladder cancer, biliary tract cancer, pancreatic cancer and colorectal cancer. Although some studies have reported drugs or components with therapeutic potential for gastrointestinal cancer, such as allicin and curcumin [2, 5]. However, due to the challenge for clinical translation of current studies and the strong aggressiveness and metastatic ability of digestive system tumors, early diagnosis and surgery are crucial for reducing the mortality and prolonging the survival time of patients with digestive system tumors [5–7]. Therefore, the identification of new potential diagnostic and prognostic tumor biomarker is helpful for the early prevention and treatment of digestive system tumors.

Non-coding RNAs including long non-coding RNAs (lncRNAs), circular RNAs (circRNAs), and microRNAs (miRNAs) are responsible for the regulation of many cells signaling pathways [8]. Moreover, there are several studies suggesting that circRNAs, lncRNAs and miRNAs are epigenetic regulators with prognostic and therapeutic effects in digestive system tumors [9–11]. LncRNAs are defined as nonprotein-coding RNAs with lengths exceeding 200 nucleotides [12, 13]. Recent studies on formation and functional mechanisms of lncRNAs have shown that lncRNAs play a key role in regulating chromatin dynamics, gene expression and maintaining biological processes [14–16]. More and more evidences indicate that the dysregulation of lncRNA expression is closely related to the development of several human diseases, such as diabetes, sepsis, stroke, autoimmune diseases and cancer [8, 17–21]. Studies on tumors have shown that lncRNAs are involved in the pathogenesis of digestive system tumors through the regulation of autophagy [1]. Therefore, lncRNAs can be used as biomarkers and therapeutic targets for cancers.

Highly up-regulated in liver cancer (HULC) is located on the chromosome 6p24.3 and approximately 500nt in length [22]. In 2007, it was first reported to be significantly up-regulated in hepatocellular carcinoma, then, increasing studies have verified that it is dysregulated in various tumors such as pancreatic cancer, breast cancer and bladder cancer [23–26]. Recently, studies have shown that HULC is overexpressed in digestive system tumors to promote tumor development [24, 27, 28]. Therefore, we believe that high expression of lncRNA HULC in patients with digestive system tumors tend to have a poor prognosis.

Meta-analysis is an analytical method that could aggregate different studies to address deficiencies caused by small sample sizes and certain human errors. Although there have been studies evaluating the prognostic value of HULC in digestive system tumors through subgroup analysis [29]. In recent years, there have been several newly-published in this field. We conducted an updated and comprehensive meta-analysis of all published studies to provide more reliable evidence to evaluate the association between HULC and the prognosis and clinicopathological features in patients with digestive system tumors.

## MATERIALS AND METHODS

### Literature retrieval strategies and selection criteria

Articles for inclusion in this meta-analysis were searched in PubMed, Web of Science and Embase, up to February 2021. The keywords used in our literature search contained (“HULC” or “lncRNA HULC”) and (“colorectal” or “gastric” or “esophageal” or “small intestine” or “hepatocellular carcinoma” or “gallbladder” or “biliary tract” or “pancreatic” or “liver” or “colon” or “rectal”). The inclusion and exclusion of the literatures were independently identified by two researchers.

### Inclusion and exclusion criteria

### Inclusion criteria

(1) The expression of lncRNA HULC in tumor tissues, serum and plasma of patients was measured; (2) According to the expression levels of HULC, patients were divided into high expression group and low expression group; (3) All patients suffered from digestive system tumors; (4) Studies were to investigate the role of HULC in digestive system tumors; (5) Survival information of patients, such as overall survival (OS), disease-free survival rate (DFS), progression-free survival rate (PFS), was provided; (6) The odds ratio (OR) or hazard ratio (HR), and the corresponding 95% confidence interval (CI) could be calculated; (6) If there were repeated studies, the latest literature was included.

### Exclusion criteria

(1) The study subjects were non-human; (2) Case reports, comments, reviews, letters and conference reports; (3) Non-English research; (4) Studies with insufficient clinical data; (5) Duplicate data or research.

### Quality assessment and data extraction

The quality assessment and data extraction of the eligible studies were conducted by two researchers independently. A third researcher was used to resolve disagreements in eligibility, data extraction, or quality assessment. The Cochrane Non-Randomized Studies Methods Group recommended the use of the Newcastle-Ottawa Scale (NOS) to assess the quality of eligible studies (http://www.ohri.ca/programs/clinical_epidemiology/oxford.asp) [30]. Publications with scores ≥ 6 were included in this meta-analysis.

The following data were extracted: (1) The first author and year of publication; (2) Tumor types and detection methods; (3) demographic characteristics, including sample size, region, age, gender and follow-up time; (4) clinical characteristics of patients, including number of tumors, TNM stage, differentiation, lymph node metastasis (LNM) and distant metastasis (DM); (5) HR with 95% CI for OS, DFS and PFS. If only Kaplan-Meier survival curves were available, the Engauge Digitizer v11.1 software could be used to obtain the available data to calculate the HR and the corresponding 95% CI [31].

### Data mining from the TCGA and GTEx data set

RNA-seq data and OS data for HULC in The Cancer Genome Atlas (TCGA) and The Genotype-Tissue Expression (GTEx) were extracted from GEPIA2 (http://gepia2.cancer-pku.cn/#index) [32]. The median cutoff was chosen to divide the patients into two groups of high and low, and plot a Kaplan–Meier curve. p < 0.05 was considered to be of prognostic value.

### Statistical analysis

Stata SE14.0 software was used for statistical analysis in this meta-analysis. Heterogeneity among all included studies was assessed by I^2^ statistics and Q test. I^2^ > 50%, P < 0.05 indicated that the heterogeneity was statistically significant. Random-effects model should be applied to improve statistical stability. I^2^ < 50%, P > 0.05 indicated no statistical significance in heterogeneity, so the fixed-effects model was used. Data extracted from Kaplan-Meier survival curves and univariate analysis data were used for pooled analysis. The relationship between the expression level of HULC and the prognosis of patients with digestive system tumors was determined by HR and the corresponding 95% CI. Also, the relationship between clinicopathological characteristics and HULC was assessed by OR and 95% CI. We used subgroup analysis to analyze the sources of heterogeneity. Sensitivity analysis was used to assess the robustness of the meta-analysis. The Egger’s test was used to evaluate the potential publication bias. HR > 1 indicated a poor prognosis. *P* value was calculated by two-tailed test, *P* < 0.05 suggested statistically significant difference.

## RESULTS

### Characteristics of eligible publications

**Figure 1:**
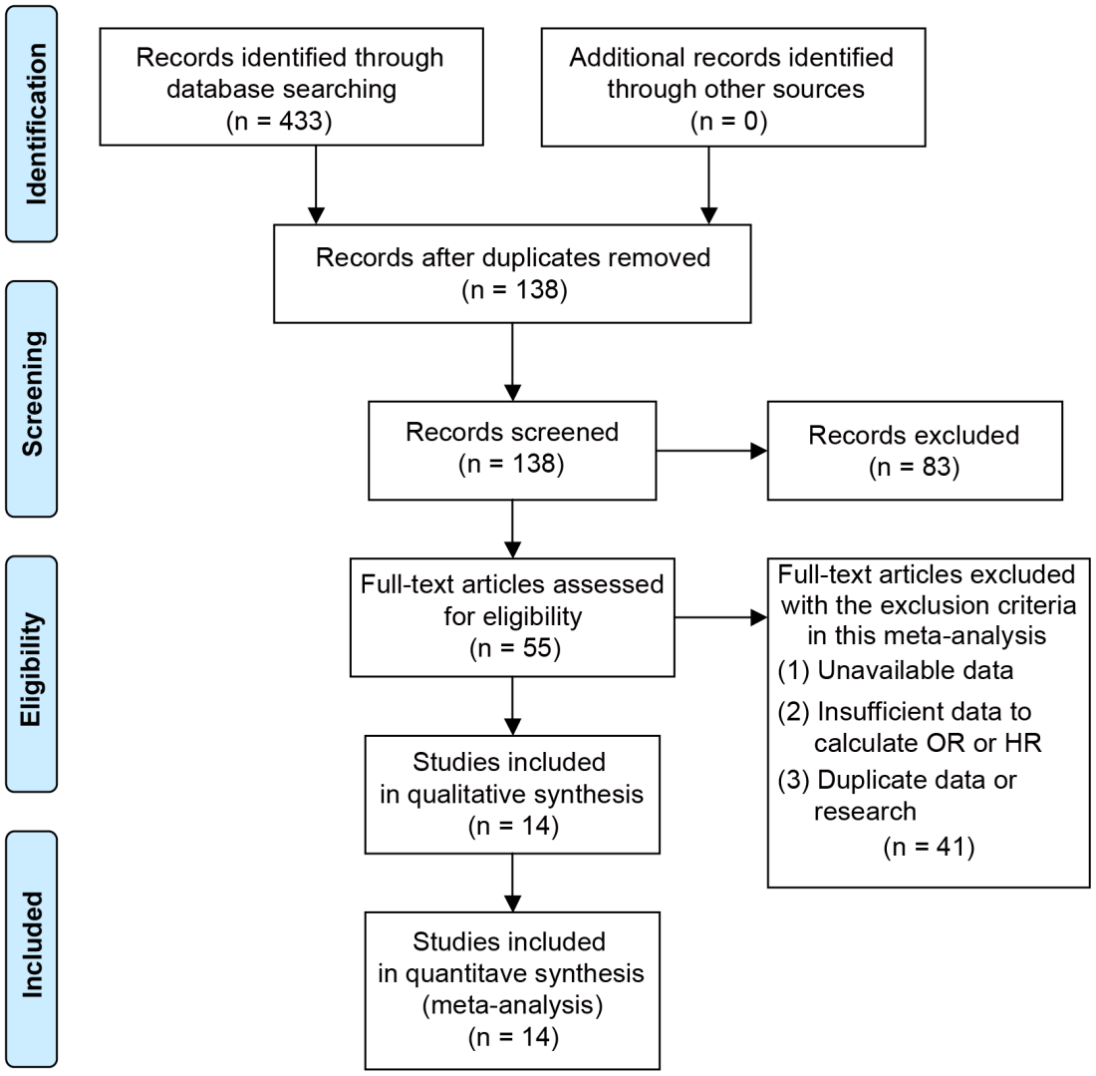
Flowchart for the process of search, selection and exclusion of studies.

Figure 1 showed the process of literature selection. After excluding duplicate studies, a total of 138 studies were obtained from PubMed, Web of Science and Embase. 83 research articles were excluded based on their titles and abstracts. After carefully reviewing the contents of the remaining 55 studies, a total of 14 articles met the inclusion criteria of this meta-analysis [24, 28, 33–44]. These studies were published between 2014 and 2020. There were 6 different kinds of digestive system tumors, including pancreatic cancer (PC, n=2), hepatocellular carcinoma (HCC, n=5), gastric cancer (GC, n=3) and colorectal cancer (CRC, n =4), in which CRC included colon cancer (CC, n=1) and colon adenocarcinoma (CA, n=1).

Table 1 summarized the main characteristics of all eligible studies in this meta-analysis. Four different regions were included in these 14 studies, China (n=11), Japan (n=1), South Korea (n=1) and Germany (n=1). A total of 1312 patients were included in this study. The sample size was a minimum of 30 and a maximum of 304. There were 5 studies with a sample size exceeding 100. In 13 studies, the HULC expression levels were determined through quantitative real-time polymerase chain reaction (qRT-PCR). In 1 study, beadchip was used to detect the HULC expression. Nine studies reported the relationship between the expression level of HULC and the clinicopathological characteristics of patients, such as age, gender, tumor size, TNM stage and tumor differentiation. The NOS scores of all included studies were ≥ 6.

**Table 1:** Basic characteristics of all qualified studies.

**Study**	**Region**	**Cancer**	**Sample size**	**Gender**		**Specimen**	**Method**	**Survival analysis**	**Outcome**	**Cut-off**	**Follow-up (months)**	**NOS**
				**F**	**M**							
Peng et al. 2014	China	PC	304	136	168	tissue	qRT-PCR	Multivariate/Univariable	OS	mean	36	8
Yang et al. 2015	Korea	HCC	240			tissue	beadchip	Multivariate/Univariable	OS/DFS	median	120	9
Li et al. 2015	China	HCC	66	13	53	plasma	qRT-PCR			median		6
Li et al. 2016	China	HCC	38	9	29	tissue	qRT-PCR	Kaplan-Meier curve	OS	mean	60	7
Jin et al. 2016	China	GC	100	35	65	serum	qRT-PCR	Kaplan-Meier curve	OS	median	36	7
Yang et al. 2016	China	CRC	35			tissue	qRT-PCR	Kaplan-Meier curve	OS	mean	120	6
Zhang et al. 2016	China	GC	42			plasma	qRT-PCR	Kaplan-Meier curve	OS	mean	60	8
Sonohara et al. 2017	Japan	HCC	158	26	132	tissue	qRT-PCR	Kaplan-Meier curve	OS/RFS	mean	120	8
Zhang et al. 2018	China	CA	50	27	23	tissue	qRT-PCR			median		6
Dong et al. 2019	China	CC	67	28	39	tissue	qRT-PCR	Kaplan-Meier curve	OS	mean	60	7
Oehme et al. 2019	Germany	CRC	52	19	33	serum exosome	qRT-PCR	Kaplan-Meier curve	OS	median	100	6
Cao et al. 2019	China	HCC	30	21	9	tissue, serum exosome	qRT-PCR			mean		6
Liu et al. 2020	China	GC	116	40	76	tissue	qRT-PCR			median		7
Ou et al. 2020	China	PC	60	17	43	serum	qRT-PCR	Multivariate/Univariable	OS	median	60	9

PC, pancreatic cancer; HCC, hepatocellular carcinoma; GC, gastric cancer; CRC, colorectal cancer; CC, colon cancer; CA, colon adenocarcinoma; F, female; M, male; OS, overall survival; DFS, disease-free survival; RFS, recurrence-free survival.

### The relationship between the expression of lncRNA HULC and OS

**Figure 2:**
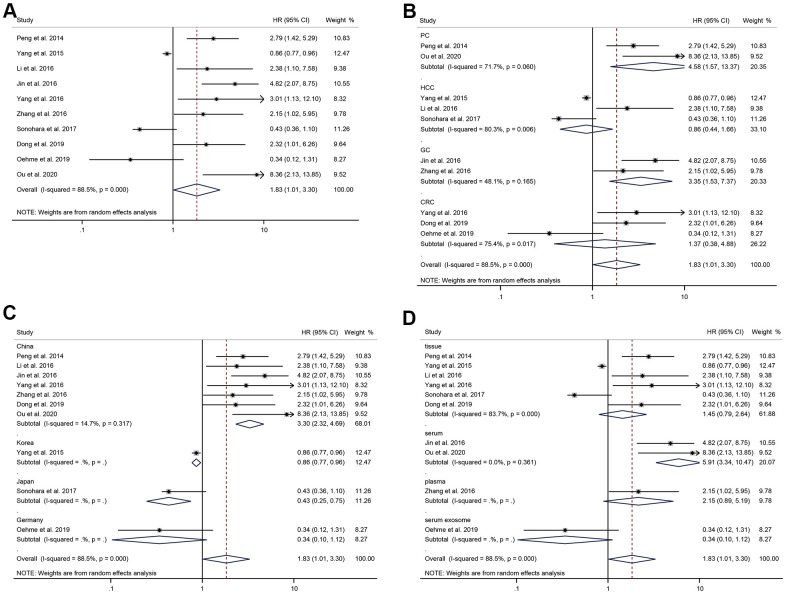
**The correlation analysis between HULC expression level and OS.** (**A**) Forest plot of the correlation between HULC expression level and OS in patients with digestive system tumors. (**B**) Subgroup analysis of HRs of OS according to the cancer type. (**C**) Subgroup analysis of HRs of OS according to the regions. (**D**) Subgroup analysis of HRs of OS according to the specimens.

There were 10 studies, including 1050 patients with digestive system tumors, reported the correlation between HULC and OS. Due to the significant heterogeneity, a random-effects model was applied to calculate the pooled HR and the corresponding 95% CI (I^2^ = 88.5%, *P* < 0.05). The results indicated that the OS of patients with up-regulated HULC expression had a worse prognosis than that of those with low HULC expression (HR = 1.83, 95% CI: 1.01-3.30, *P* = 0.045) (Figure 2A).

In term of cancer type, high expression of HULC was significantly related to poor survival prognosis in PC (HR = 4.58, 95%CI: 1.57-13.37, P = 0.005) with significant heterogeneity (I^2^ = 71.7%, P = 0.060), and in GC (HR = 3.35, 95%CI: 1.53-7.37, P = 0.003) with no significant heterogeneity (I^2^ = 48.1%, P = 0.165) (Figure 2B). There was no significant correlation between HULC expression level and prognosis in HCC (HR = 0.86, 95%CI: 0.44-1.66, P = 0.65) with significant heterogeneity (I^2^ = 80.3%, P = 0.006), and in CRC (HR = 1.37, 95%CI: 0.38-4.88, P = 0.63) with significant heterogeneity (I^2^ = 75.4%, P = 0.017). According to subgroup analyses stratified by regions, the four geographic locations presented inconsistent results. In China, higher HULC expression level was related to poor prognosis (HR = 3.30, 95%CI: 2.32-4.69, P < 0.001) with no significant heterogeneity (I^2^ = 14.7%, P = 0.32). However, it was associated with favorable prognosis in Japan (HR = 0.43, 95%CI: 0.25-0.75, P = 0.003) and Korea (HR = 0.86, 95%CI: 0.77-0.96, P = 0.007). In Germany, the association between HULC expression level and prognosis of digestive system tumors was not statistically significant (HR =0.34, 95%CI: 0.10-1.12, P = 0.077) (Figure 2C). When the studies were stratified by specimens, we found that the result of serum was statistically significant (HR = 5.91, 95%CI: 3.34-10.47, P < 0.001) with significant heterogeneity (I^2^ = 0.0%, P = 0.361); nevertheless, the significant association was not found in tissue (HR = 1.45, 95%CI: 0.79-2.64, P = 0.23) with significant heterogeneity (I^2^ = 83.7%, P < 0.001), plasma (HR = 2.15, 95%CI: 0.89-5.19, P = 0.089) or serum exosomes (HR = 0.34, 95%CI: 0.10-1.12, P = 0.077) (Figure 2D). The above results suggested that cancer type and region were the sources of heterogeneity in this meta-analysis.

**Figure 3:**
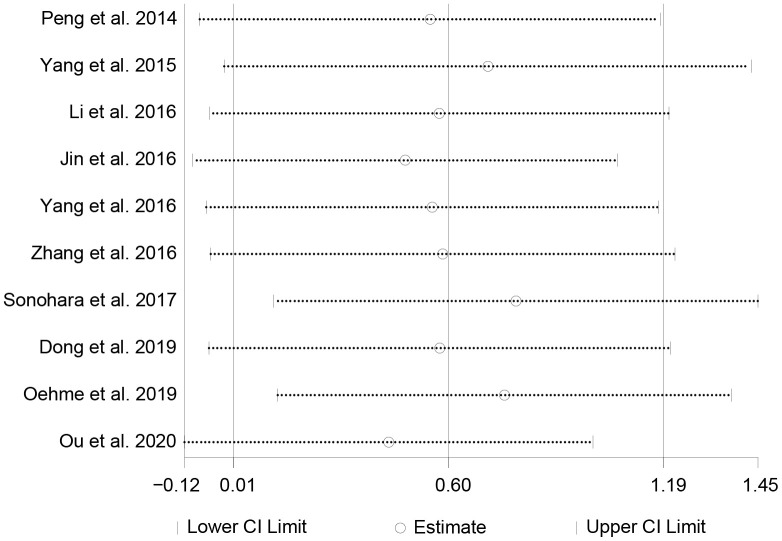
The sensitivity analysis on the correlation between HULC expression level and OS.

Sensitivity analysis observed whether the pooled results were affected by eliminating each study in turn. Figure 3 showed the results of sensitivity analysis, which suggested that the results were reliable.

### The relationship between the expression of HULC and tumor size, number, TNM stage, differentiation

**Figure 4:**
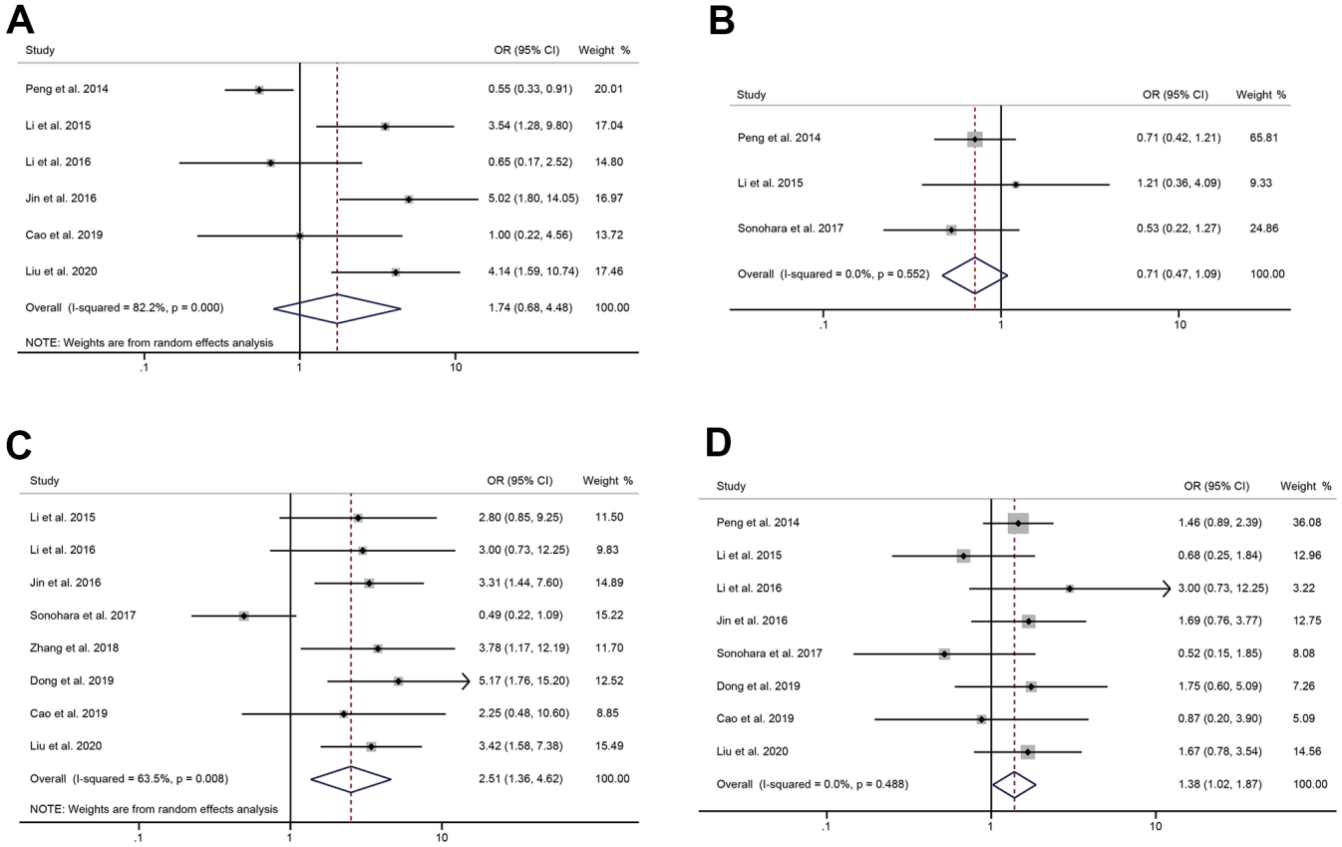
**The correlation between HULC expression level and tumor characteristics.** (**A**) The forest plot of ORs for the correlation between HULC expression and tumor size in patients with digestive system tumors; (**B**) The forest plot of ORs for the correlation between HULC expression and number of tumors in patients with digestive system tumors; (**C**) The forest plot of ORs for the correlation between HULC expression and TNM stage in patients with digestive system tumors; (**D**) The forest plot of ORs for the correlation between HULC expression and differentiation in patients with digestive system tumors.

There were 6 and 3 studies respectively reporting the tumor size and number of tumors in patients with digestive system tumors. The pooled results showed that the expression level of HULC was not correlated to tumor size (OR = 1.74, 95%CI: 0.68-4.48, *P* = 0.25) or number of tumors (OR = 0.71, 95%CI: 0.47-1.09, *P* = 0.11) (Figure 4A, 4B). A random-effects model was used to analyze the relationship between HULC expression and TNM stage (I^2^ = 63.5%, *P* = 0.008). The pooled OR was 2.51 (95%CI: 1.36-4.62, *P* = 0.003), suggesting a significant correlation between the expression of HULC and TNM stage (Figure 4C). Thus, overexpression of HULC could easily increase the risk of high-stage tumors. Eight of included studies reported the differentiation of tumors. Since there was no significant heterogeneity, we used a fixed-effects model (I^2^ = 0.0%, *P* = 0.488) to analyze the data of tumor differentiation. The results suggested that the increased expression of HULC was significantly related to the poor differentiation of the digestive system tumors (OR = 1.38, 95% CI: 1.02-1.87, *P* = 0.035) (Figure 4D).

### The relationship between the expression of HULC and LNM, DM

**Figure 5:**
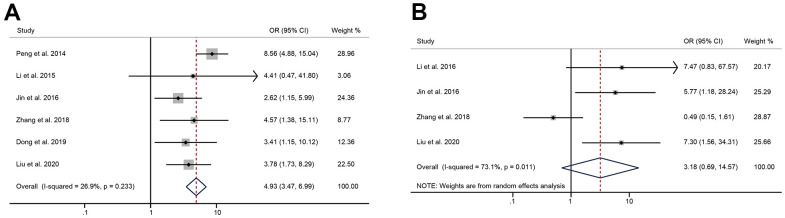
**The correlation between HULC expression level and metastasis.** (**A**) The forest plot of ORs for the correlation between HULC expression and lymphatic node metastasis in patients with digestive system tumors; (**B**) The forest plot of ORs for the correlation between HULC expression and distant metastasis in patients with digestive system tumors.

A total of 6 qualified literatures reported the occurrence of LNM in patients with digestive system tumors. Since no significant heterogeneity was found, we adopted a fixed-effects model (I^2^ = 26.9%, *P* = 0.233). The pooled result indicated that cancer patients with high HULC expression had higher risk of LNM than those with low HULC expression (OR = 4.93, 95% CI: 3.47-6.99, *P* < 0.001) (Figure 5A). I^2^ = 73.1%, *P* = 0.011 suggested significant heterogeneity, so a random-effects model was used for the pooled analysis of DM. We found that high HULC expression was not significantly correlated with the DM of tumors (OR = 3.18, 95% CI: 0.69-14.57, *P* = 0.14) (Figure 5B).

### The relationship between the expression of HULC and patients’ age, gender

**Figure 6:**
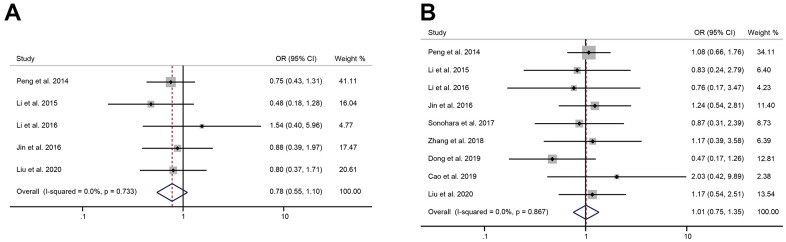
**The correlation between HULC expression level and the patient’s age, gender.** (**A**) The forest plot of ORs for the correlation between HULC expression and age in patients with digestive system tumors; (**B**) The forest plot of ORs for the correlation between HULC expression and gender in patients with digestive system tumors.

In 5 studies (1 on pancreatic cancer, 2 on hepatocellular carcinoma, and 2 on gastric cancer), the high expression of HULC was not significantly related to the age of patients (OR = 0.78, 95% CI: 0.55-1.10, *P* = 0.16) (Figure 6A). The gender of patients was mentioned in 9 qualified studies. Since the heterogeneity was not significant, we used a fixed-effects model (I^2^ = 0.0%, *P* = 0.867). The pooled OR was 1.01 (95% CI: 0.75-1.35, *P* = 0.95), indicating that the overexpression of HULC was not significantly associated with the gender of patients (Figure 6B).

### Assessment of publication bias

**Figure 7:**
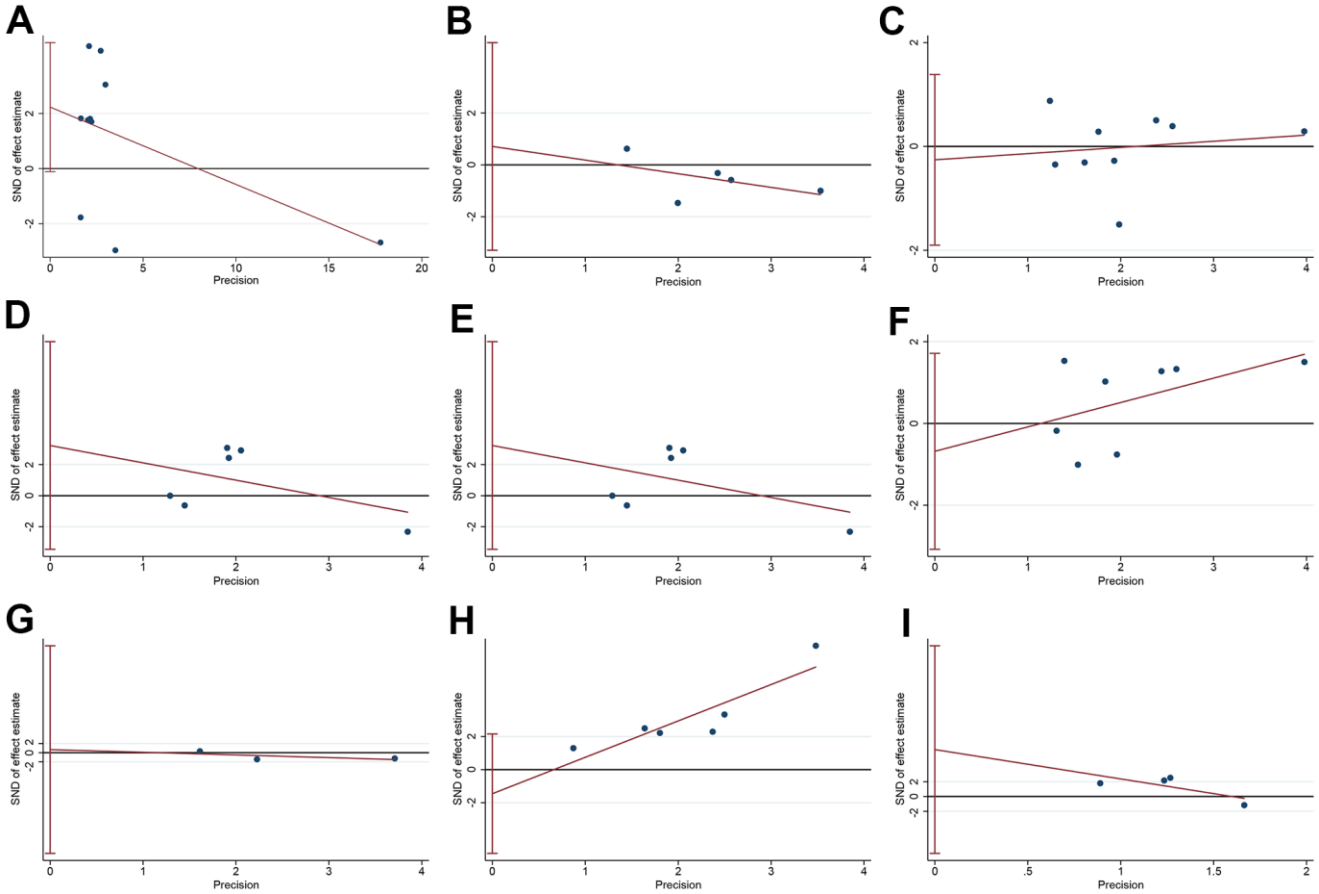
**The publication bias of HULC-related research.** (**A**) The Egger’s test and linear regression plot for the publication bias of OS; (**B**) The Egger’s test and linear regression plot for the publication bias of age; (**C**) The Egger’s test and linear regression plot for the publication bias of gender; (**D**) The Egger’s test and linear regression plot for the publication bias of tumor size; (**E**) The Egger’s test and linear regression plot for the publication bias of TNM stage; (**F**) The Egger’s test and linear regression plot for the publication bias of differentiation; (**G**) The Egger’s test and linear regression plot for the publication bias of number of tumors; (**H**) The Egger’s test and linear regression plot for the publication bias of lymphatic node metastasis; (**I**) The Egger’s test and linear regression plot for the publication bias of distant metastasis.

To assess the publication bias in the current study, we performed an Egger’s linear regression test. The Egger’s test and linear regression plot was shown in Figure 7. Moreover, there was no statistically significant publication bias in OS (*P* = 0.059), age (*P* = 0.61), gender (*P* = 0.72), tumor size (*P* = 0.25), TNM stage (*P* = 0.25), differentiation (*P* = 0.51), number of tumors (*P* = 0.77), LNM (*P* = 0.33) and DM (*P* = 0.19).

### Results in TCGA and GTEx data set

**Figure 8:**
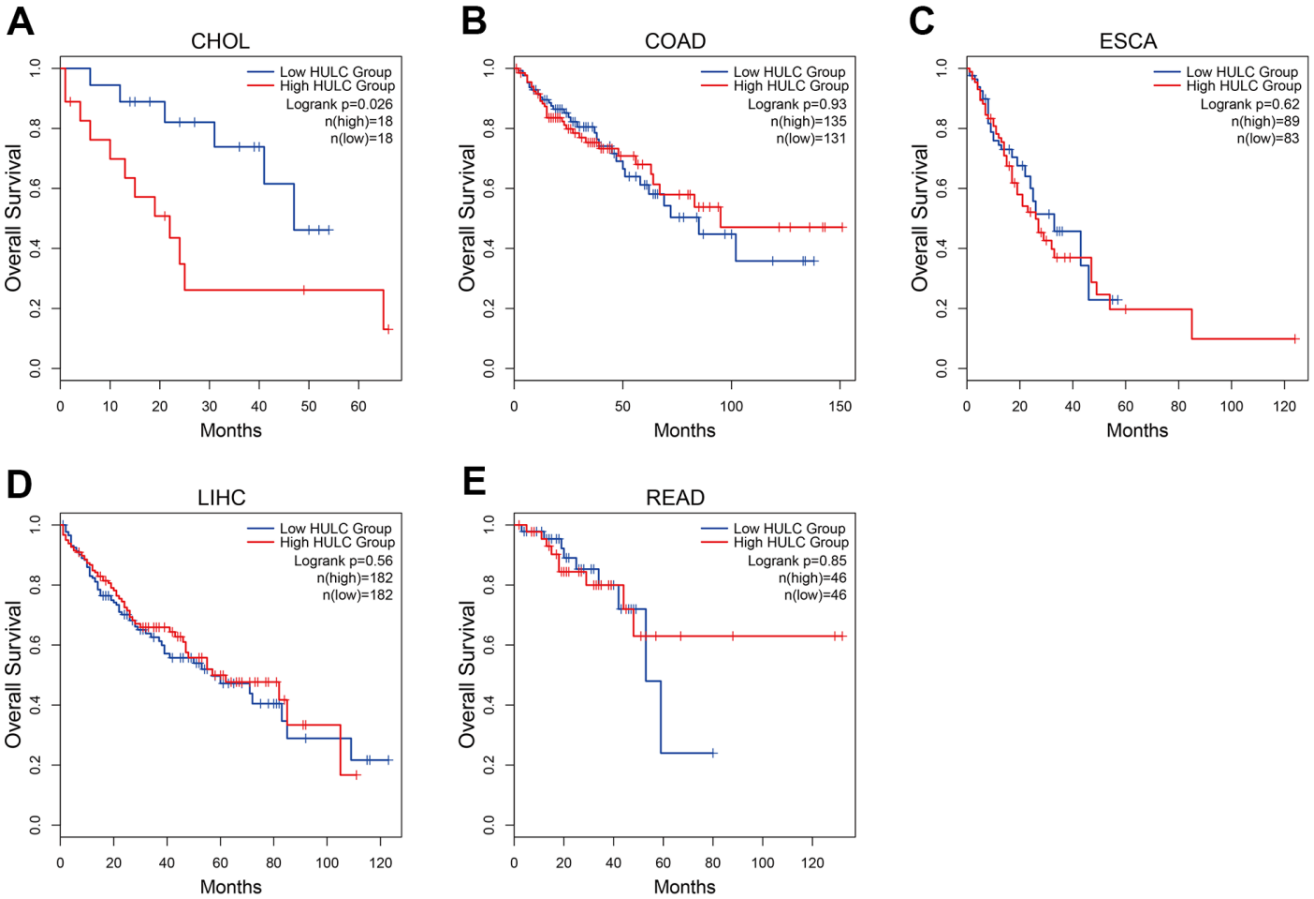
**Kaplan–Meier curves showing the prognostic value of HULC in TCGA and GTEx data set.** (**A**) The Kaplan–Meier curve of CHOL; (**B**) The Kaplan–Meier curve of COAD; (**C**) The Kaplan–Meier curve of ESCA; (**D**) The Kaplan–Meier curve of LIHC; (**E**) The Kaplan–Meier curve of READ. CHOL: cholangiocarcinoma; COAD: colon adenocarcinoma; ESCA: esophageal carcinoma; LIHC: liver hepatocellular carcinoma; READ: rectum adenocarcinoma.

The prognostic value of HULC was further verified by retrieving the clinical data from TCGA and GTEX data set. We finally retrieved 5 GI cancers, including cholangiocarcinoma (CHOL), colon adenocarcinoma (COAD), esophageal carcinoma (ESCA), liver hepatocellular carcinoma (LIHC) and rectum adenocarcinoma (READ) (Figure 8). Unexpectedly, the high expression of HULC was negatively correlated with OS time in CHOL, while HULC expression was not significantly correlated with OS time in other cancers. Moreover, CHOL was a kind of GI cancers not included in our meta-analysis.

## DISCUSSION

Because lncRNA can regulate gene expression through epigenetic modification, transcription and post-transcriptional translation, lncRNAs have key functions in various diseases [45]. It has been found that lncRNAs are critical for angiogenesis and neuroprotection. Therefore, lncRNAs were considered as therapeutic, diagnostic and prognostic tools in cerebrovascular diseases, including stroke [17]. Recently, lncRNA has been proved to be a key factor in tumorigenesis, and it can involve in cancer by regulating tumor cell proliferation, migration and DNA stability [46]. In gynecological cancer, lncRNA is considered as a biomarker or therapeutic target [20]. LncRNAs such as MALAT1, XIST and NORAD have been proven to be biomarkers for human tumor prognosis [47–49]. Also, some studies have shown that HULC expression is dysregulated in digestive system tumors [37, 39, 50]. Therefore, we believe that HULC can be used as a new potential diagnostic and prognostic tumor biomarker for digestive system tumors.

We conducted this meta-analysis to verify the correlation between HULC expression level and survival prognosis of patients with digestive system tumors. A total of 14 studies involving 1312 patients were included. The pooled results suggested that high expression level of HULC had a significant correlation with poor survival prognosis in patients with digestive system tumors. Sensitivity analysis showed that our analysis was robust. We found that high expression level of HULC could increase the risk of high TNM stage, poor differentiation, and LNM. HULC expression level was not associated with age, gender, tumor size, number of tumors, or DM. The results of subgroup analysis suggested that cancer type and region were the sources of heterogeneity in this meta-analysis. There was no significant publication bias among all included studies. These results suggested that HULC may be a candidate oncogene for digestive system tumors. The up-regulated of HULC could be used as a novel predictor of poor prognosis in patients with digestive system tumors.

Since most studies prefer to report positive results rather than negative results. We performed a further verification for prognostic value of HULC in patients with digestive system tumors. By analyzing TCGA and GTEX data set, we found unexpected results. The high expression of HULC was negatively correlated with OS time in CHOL, while HULC expression was not significantly correlated with OS time in other cancers. This finding deserves further investigation.

According to previous reports, HULC can exert oncogenic effect through different mechanisms. Many cancer researchers have made great efforts to explore the functional mechanism of lncRNA HULC on the occurrence and development of various cancers. YB-1 is a multifunctional protein that participates in cellular functions, such as transcription regulation, mRNA splicing and translation regulation [51, 52]. It has been reported that HULC can promote the phosphorylation of YB-1 protein to activate the translation of silent oncogenes, so as to promote the occurrence of hepatocellular carcinoma [53]. The “Warburg effect” refers to the reprogramming of glucose metabolism from oxidative phosphorylation to aerobic glycolysis, which is one of the hallmarks of tumor cells [54]. Wang et al. have found that HULC can enhance the binding of LDHA and PKM2 to FGFR1, resulting in increased phosphorylation of these two enzymes and consequently promoting glycolysis [55]. Non-coding RNAs (ncRNAs) can transfer information from their donor cells to recipient cells through exosomes to play a role in cell-to-cell communication [56]. Circulating extracellular vesicle-encapsulated HULC showed good predictive performance in distinguishing pancreatic ductal adenocarcinoma (PDAC) [57]. Takahashi et al. 2020 et al. reported that miR-622 encapsulated by exosomes can inhibit epithelial-mesenchymal transition (EMT) by targeting HULC to inhibit the invasion and migration of pancreatic ductal adenocarcinoma cells [58]. Autophagy is recognized an intracellular regulatory process [10]. There is evidence that autophagy plays an important role in both progression and suppression of digestive system tumors [1, 10]. Thus, HULC can not only increase the expression of P62 by reducing mature miR-15a, but also increase autophagy by increasing Sirt1-dependent LC3II to promote the development of hepatocellular carcinoma [59]. STAT3, a transcription factor involved in immune response, inflammation and tumorigenesis, has been found to be critical for compensatory liver regeneration and chemically-induced HCC development [60]. Liu et al. indicated that HULC can elevate HBx, which co-activated the STAT3 to stimulate the miR-539 promoter, and then down regulated APOBEC3B to activate HBV in HBV-related hepatocellular carcinoma [61]. The expression of HULC and endothelial cell specific molecule 1 (ESM-1) in glioma tissue is positively correlated with microvessel density and hierarchical dependence. Thus, the pro-angiogenic activity mechanism of HULC may be achieved by regulating ESM-1 through the PI3K/ Akt/ mTOR signal transduction pathway [62].

We found several meta-analyses evaluating the correlation between HULC expression level and prognosis. However, there is some difference between our study and the previous meta-analyses. First, the inclusion criteria are different. The previously meta-analyses evaluated the correlation between HULC expression and survival prognosis of cancer patients. These meta-analyses included various cancers. However, our study only included patients with digestive system tumors. A meta-analysis by Li et al. has no restrictions on the language of the publications [63]. Secondly, the number of eligible studies is increased significantly. The publications related to digestive system tumors included in the previous meta-analyses was 4-6, while we included 14 studies reporting digestive system tumors [29, 63–66]. Third, previous studies do not evaluate the correlation between HULC expression levels and tumor differentiation. Our meta-analysis confirms the association between HULC expression levels and survival prognosis in patients with digestive system tumors, and adds the analysis of tumor size, number of tumors, differentiation, age and gender of patients. Finally, although most of the studies were from China, this meta-analysis also included studies from South Korea, Japan and Germany.

However, this study still has some obvious limitations. First, Due to the complexity of digestive cancer types, the mechanism of HULC may be different among digestive system tumors. Then, there was no uniform cut-off value to define high HULC expression and low HULC expression. All included studies divided patients into high HULC expression group and low HULC expression group by mean or median. Third, the limited number of eligible studies and data in the analyses, leading to low statistical power and incomplete results. The HR and 95% CI extracted from the Kaplan-Meier curve were far less reliable than the values directly provided by original studies. Since the extracted HR and 95%CI could be affected by the subjective factors, it may lead to deviations of calculation. Next, the heterogeneity could not be completely improved, though the results of sensitivity analysis showed that our results were relatively robust. Finally, most of the included studies were from China, and the rest came from South Korea, Japan, and Germany. This limited the results of the study by region and ethnicity. Therefore, the results of this meta-analysis should be treated with caution for other ethnic groups.

In conclusion, this meta-analysis confirms that patients with up-regulated HULC may cause poorer clinical outcomes. Our results also show that the high expression level of HULC in serum and plasma was related to poor survival prognosis. HULC, especially from serum and plasma, can be used as a new potential predictor of the prognosis of patients with digestive system tumors. High expression of HULC increases the risk of high tumor stage, poor differentiation, and LNM in digestive system tumors. In view of the limitations mentioned above, it is necessary to conduct a more carefully designed study with diverse ethnic groups and large sample sizes to confirm the results of this meta-analysis and determine the predictive value of HULC in the prognosis of digestive system tumors.

### Ethics approval and consent to participate

Ethical approval was not needed because this is a meta-analysis.

### Availability of data and material

The datasets used and/or analysed during the current study are available from the corresponding author on reasonable request.
